# A Key Role for Poly(ADP-Ribose) Polymerase 3 in Ectodermal Specification and Neural Crest Development

**DOI:** 10.1371/journal.pone.0015834

**Published:** 2011-01-17

**Authors:** Michèle Rouleau, Vishal Saxena, Amélie Rodrigue, Eric R. Paquet, Abbie Gagnon, Michael J. Hendzel, Jean-Yves Masson, Marc Ekker, Guy G. Poirier

**Affiliations:** 1 Cancer Research Laboratory, CHUQ Research Center, Centre Hospitalier de l'Université Laval, Québec, Canada; 2 Department of Molecular Biology, Medical Biochemistry and Pathology, Faculty of Medicine, Laval University, Québec, Canada; 3 Center for Advanced Research in Environmental Genomics, Department of Biology, University of Ottawa, Ottawa, Canada; 4 Genome Stability Laboratory, Laval University Cancer Research Center, Hôtel-Dieu de Québec, Québec, Canada; 5 Department of Oncology, Faculty of Medicine, University of Alberta and Cross Cancer Institute, Edmonton, Canada; Ohio State University, United States of America

## Abstract

**Background:**

The PARP family member poly(ADP-ribose) polymerase 3 (PARP3) is structurally related to the well characterized PARP1 that orchestrates cellular responses to DNA strand breaks and cell death by the synthesis of poly(ADP-ribose). In contrast to PARP1 and PARP2, the functions of PARP3 are undefined. Here, we reveal critical functions for PARP3 during vertebrate development.

**Principal Findings:**

We have used several *in vitro* and *in vivo* approaches to examine the possible functions of PARP3 as a transcriptional regulator, a function suggested from its previously reported association with several Polycomb group (PcG) proteins. We demonstrate that PARP3 gene occupancy in the human neuroblastoma cell line SK-N-SH occurs preferentially with developmental genes regulating cell fate specification, tissue patterning, craniofacial development and neurogenesis. Addressing the significance of this association during zebrafish development, we show that morpholino oligonucleotide-directed inhibition of *parp3* expression in zebrafish impairs the expression of the neural crest cell specifier *sox9a* and of *dlx3b*/*dlx4b*, the formation of cranial sensory placodes, inner ears and pectoral fins. It delays pigmentation and severely impedes the development of the median fin fold and tail bud.

**Conclusion:**

Our findings demonstrate that Parp3 is crucial in the early stages of zebrafish development, possibly by exerting its transcriptional regulatory functions as early as during the specification of the neural plate border.

## Introduction

Poly(ADP-ribose) polymerase-3 (PARP3) belongs to the small family of PARP enzymes that catalyze the poly(ADP-ribosyl)ation of protein substrates. PARP3 is structurally related to the well studied PARP1, a modulator of chromatin structure that plays important roles in the DNA damage response, cell death signaling and transcriptional regulation, and to PARP2, that is also involved in the maintenance of genomic integrity (reviewed in [1], [2]). Poly(ADP-ribosyl)ation is a transient post-translational modification that affects mostly chromatin-related proteins. The transient nature of poly(ADP-ribosyl)ation is conferred by poly(ADP-ribose) glycohydrolase, an enzyme that specifically and rapidly hydrolyzes poly(ADP-ribose) shortly after synthesis. In spite of its identification more than 10 years ago [3], the functions of PARP3 are poorly known. The association of PARP3 with PARP1 and several DNA damage repair proteins, its significant activation by DNA strand breaks in vitro, and its ability to facilitate repair by non-homologous end-joining indicate that PARP3 is a constituent of the DNA damage response [4], [5], [6]. However, in comparison to PARP1 and PARP2 which are highly stimulated by DNA strand breaks, the poly(ADP-ribose) polymerase activity of PARP3 appears modest [6]. While PARP2 partially compensates for the reduced or absent expression of PARP1 during mouse development, PARP3 cannot effectively compensate for the absence of PARP1 and/or PARP2 [7], [8]. Furthermore, the tissue distribution of PARP1 and PARP2 which overlaps to great extents in mammals, differs significantly from the tissue distribution of PARP3 [8], [9]. Collectively, these observations suggest that PARP3 has functions distinct from those of PARP1 and PARP2, despite significant structural similarities.

We have recently reported that human PARP3 associates with several Polycomb group (PcG) proteins of the PRC2 complex, namely EZH2, SUZ12, RBAP46/48, YY1 and HDAC1/2 [5]. Together with the trithorax protein group (trxG), PcG proteins are key regulators of embryogenesis and development [10], [11]. They form protein complexes that maintain cell pluripotency by repressing the expression of differentiation genes. Two types of Polycomb repressive complexes (PRC) act in concert by marking chromatin domains with histone posttranslational modifications and by mediating chromatin compaction to repress transcription. PRC2 comprises the histone methyltransferase EZH2 that trimethylates histone H3 on lysine 27 (H3K27me3) and the core regulatory subunits SUZ12, EED and JARID2 while PRC1 comprises BMI-1 and the E3-ligase RING1B that ubiquitylates lysine 119 of histone H2A [12], [13], [14], [15]. In addition to the core PRC components, several other proteins are facultative partners, such as the histone deacetylases HDAC1/2 and the transcription factor YY1, that provide additional attributes to PRC2, such as the ability to co-regulate histone acetylation and possibly the recruitment of PRC2 to specific chromatin domains during development, respectively [16], [17].

The association of PARP3 with PcG proteins suggested that PARP3 participates in the epigenetic regulation of transcription and development. It led us to examine the functions of PARP3 during vertebrate development using zebrafish as a model system and to identify genes associated with PARP3 in the human neuroblastoma cell line SK-N-SH. We find that a significant number of genes involved in development and neuronal specification are PARP3 targets, of which several are also known PRC2 targets. An *in vivo* analysis of the expression of PARP3-bound developmental genes in the context of reduced PARP3 expression in zebrafish embryos reveals that it regulates the expression of several developmental genes critical for the specification of neural crest cells at the neural plate border of zebrafish embryos. The reduced expression of PARP3 leads to several developmental defects caused by inappropriate ectodermal and neural crest cell differentiation, indicating that it is essential for vertebrate development.

## Results

### PARP3 is essential for zebrafish development

**Figure 1:**
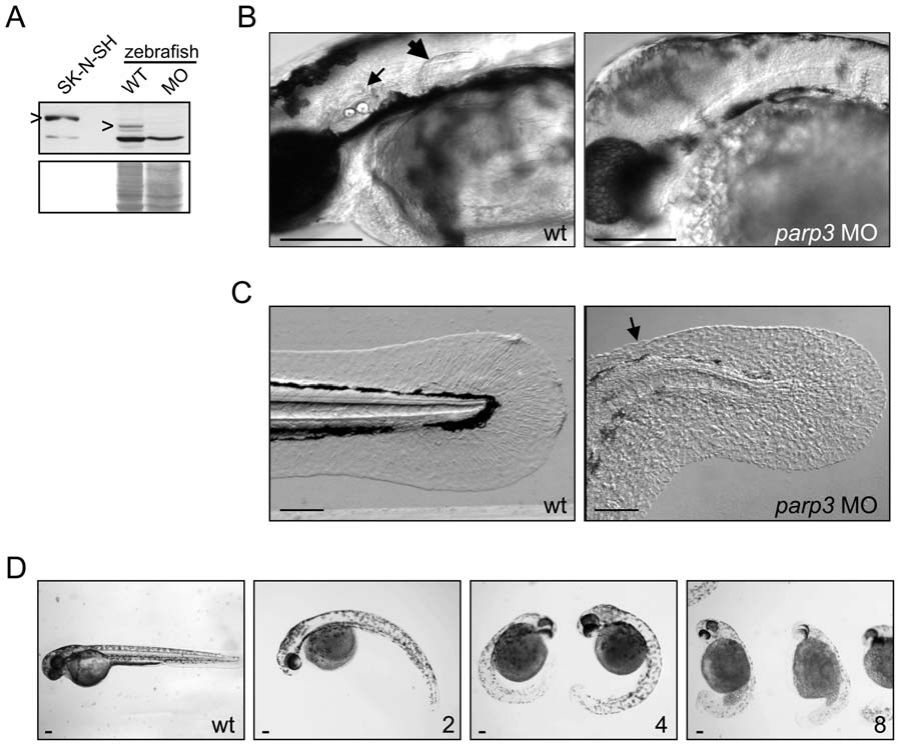
Developmental perturbations in zebrafish embryos with impaired *parp3* expression. A. Immunoblot analysis (upper panel) of zebrafish Parp3 in wild type (WT) and *parp3* morphants (MO) using an antibody raised against human PARP3. A whole cell extract of human SK-N-SH cells is shown as a control. The protein bands corresponding to PARP3 are indicated by “>”. The faster migrating band corresponds to a non-related protein that cross-reacts with the antibody. The Western blot membrane was stained with Ponceau S as a protein loading control (lower panel). B. Enlarged lateral views of the head regions of wild type and *parp3* morphants injected with 4 ng MO1. The inner ears (small arrow) and the pectoral fins (large arrow) in the wt embryos are not formed in the *parp3* morphants. C. Enlarged lateral views of the tail of wild type and *parp3* morphants injected with 4 ng MO1. The median fin fold (arrow) is less developed in the morphants and has a more granular aspect. Effects are more pronounced on the dorsal side (arrow). D. Zebrafish embryos 48hrs after injection of increasing amounts of the *parp3*-specific morpholino oligonucleotide MO1 at the one-cell stage (ng amounts indicated in the lower right corner). The short length of morphant embryos, their curved tail and their reduced pigmentation is increasingly severe with increasing amounts of injected MO1. Lateral views with anterior to the right and dorsal to the top. Scale bars represent 10 µm.

To investigate the biological functions of PARP3, we exploited the rapid and well characterized development schedule of the zebrafish. The zebrafish genome comprises a single *parp3* gene orthologous to the human *PARP3* gene. The human gene however encodes two PARP3 isoforms due to alternative splicing of the PARP3 transcripts. A long PARP3 isoform, expressed at low levels, comprises a 7 amino acid extension on its N-terminal side that is absent in the short and predominant isoform [5]. Based on an analysis of EST sequences, the zebrafish genome, similar to the mouse genome, appears to code only for the short isoform (Fig. S1A). Overall, zebrafish Parp3 shares 71% sequence similarity with the human PARP3 (short) sequence. The N-terminal domain, that lacks any similarity with known domains, is less well conserved (48% similarity) than the putative nucleic acid binding WGR domain (77% similarity) and the PARP catalytic domain (76% similarity). The catalytic core H-Y-E amino acid triad, critical for NAD^+^ binding and PARP activity, is conserved (Fig. S1A) [18]. Furthermore, antibodies raised against human PARP3 recognize zebrafish Parp3 (Fig. 1A). These observations support the notion that zebrafish Parp3 is highly related to human PARP3 at the amino acid level and that PARP3 is an evolutionarily conserved protein in multicellular organisms.

To address the biological functions of Parp3 during zebrafish development, the expression of *parp3* was knocked down by microinjection of morpholino oligonucleotides (MOs) into one-cell stage zebrafish embryos. Two non-overlapping MOs, one targeting the transcriptional start site of the *parp3* gene (MO1) and the other the 5′UTR sequence immediately upstream of the transcriptional start site (MO2) (Fig. S1B), were used to monitor the effects of reduced Parp3 levels on zebrafish development. Both MOs induced an effective knock-down of Parp3 expression (data shown for MO1, Fig. 1A) that resulted in important developmental defects and lethality by 4–6 days following fertilization (data shown for MO1 in Fig. 1B–D). Visual inspection of the zebrafish embryos revealed a motility defect 24 hours after fertilization (hpf) that remained until death of embryos. While wild type embryos and those injected with a control MO present a typical spontaneous contractile movement, embryos injected with *parp3* MOs display little movement (Movie S1). By 48 hpf, most *parp3* morphants have not hatched, lack inner ears and pectoral fin buds (Fig. 1B), and their pigmentation is drastically delayed (Fig. 1C, D). They show a highly curved trunk with very short tail (Fig. 1D). The trunk musculature of *parp3* morphants appears normal but the tail bud is ill-developed (Fig. 1C). The median fin fold of *parp3* morphants is severely affected (Fig. 1C). The symmetrical median fin fold of wild type embryos contrasts with that of morphants which is less developed and particulary perturbed on the dorsal side (Fig. 1C, arrow). The fin fold of morphants displays a granular aspect and the actinotrichia (unmineralized structural fibrils of the median fin fold) appear shorter or less clearly visible. Furthermore, the severity of the phenotype increases with the dose of injected *parp3* MO (Fig. 1D) and defects are not restored at later times post-fertilization, indicating that they are irreversible consequences of reduced Parp3 expression.

The two *parp3* MOs produced the same developmental defects, indicating that the observed phenotypes are due to the knock-down of Parp3 and not to off-target effects. Furthermore, co-injection of a *p53* MO with *parp3* MO1 or MO2 did not rescue the observed developmental defects (data not shown), eliminating the concern that *parp3* MO-associated phenotypes could be resulting from a non-specific up-regulation of p53 expression [19].

In an effort to characterize further the defects induced by the reduced expression of Parp3, we used transgenic Tg(fli1:EGFP)^y1^ zebrafish embryos to evaluate the development of the vasculature in parp3 morphants [20]. Despite the severely distorted aspect of the morphants and their ill-developed tail region, the knock-down of Parp3 expression does not impair the vascular development (Fig. S2A). Similarly, development of the floor plate (Fig. S2B) and motoneurons (Fig. S2C) are not altered by the reduced expression of Parp3, and are therefore an unlikely explanation for the impaired motility of *parp3* morphants.

Collectively, these observations indicate that the knock-down of Parp3 has pleiotrophic effects on development and suggest an important function for this protein during development.

### PARP3 is associated with chromatin and with components of Polycomb repressive complex 2

**Figure 2:**
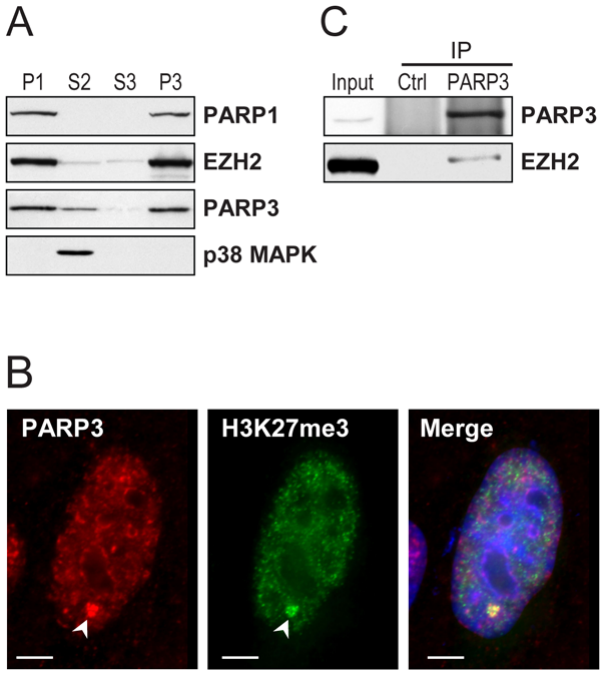
PARP3 is associated with chromatin and Polycomb proteins. A. The human neuroblastoma cell line SK-N-SH was fractionated into nuclear (P1) and cytoplasmic (S2) fractions. The nuclear P1 fraction was further separated into nuclear soluble (S3) and chromatin (P3) fractions. Proteins detected by immunoblotting are indicated. PARP1 and p38 MAPK were used as chromatin and cytoplasmic markers, respectively. Samples of each fraction correspond to an equal cell number. B. Immunofluorescence detection of PARP3 in SK-N-SH cells. PARP3 is predominantly nuclear and co-localizes with trimethylated histone H3K27 (H3K27me3) (arrowhead). Scale bars represent 3 µm. C. Immunoblot analysis of proteins immunoprecipitated (IP) with nuclear PARP3. EZH2 co-precipitates with PARP3. Input corresponds to 10% of the cellular extract used in IP. Rabbit IgG were used in the control (Ctrl) IP.

To investigate the molecular functions of PARP3 and gather some hints about how PARP3 could regulate zebrafish development, we turned to a molecular analysis of PARP3 in the human cell line SK-N-SH. In contrast to expression levels beyond detection limits in many human cell lines, PARP3 expression in the neuroblastoma cell line SK-N-SH permits a detailed cellular analysis [9]. Analysis of the subcellular distribution of PARP3 reveals a preferential accumulation in the nuclear fraction (Fig. 2A, lane P1), although some PARP3 resides in the cytoplasm (Fig. 2A, lane S2). This contrasts with the type member of the PARP family, PARP1, which is exclusively nuclear (Fig. 2A). A large proportion of nuclear PARP3 is associated with chromatin (Fig. 2A, lane P3). The cellular distribution of PARP3 assessed by immunofluorescence reveals that nuclear PARP3 accumulates in numerous small foci as well as in a few larger foci that we previously showed to correspond to Polycomb group bodies (Fig. 2B) [5]. The large foci are also enriched with histone H3 trimethylated on lysine 27 (H3K27me3) (Fig. 2B). This epigenetic mark results from the activity of the methyltransferase EZH2 in the Polycomb complex PRC2 [21]. Further indications that some PARP3 is associated with several components of the PRC2 complex such as EZH2, SUZ12 and RBAP46/48 were obtained by analysis of proteins immunoprecipitated with endogenous PARP3 (Fig. 2C) and FLAG-tagged PARP3 [5]. These observations indicate that PARP3 is a chromatin-associated protein, of which some is associated with Polycomb proteins of the PRC2 complex. Furthermore, these results suggest that PARP3 could exert regulatory functions at the transcriptional level, given its association with PRC2 and the phenotype of zebrafish morphants with reduced PARP3 expression.

### Genomic distribution of PARP3

Given that PARP3 is associated with chromatin and co-localizes with H3K27me3, a posttranslational modification that is highly correlated with genomic silencing, we carried out an investigation of the genomic occupancy of PARP3 in human SK-N-SH cells to assess the roles of PARP3 in gene transcription. Using a commercially available antibody that recognizes specifically PARP3, we pulled-down PARP3-associated sequences and conducted a ChIP-chip analysis. Control ChIP were done with rabbit IgG. Labelled PARP3 and IgG bound DNA were hybridized to an Agilent promoter array comprising probes covering regions from −5.5 kb to +2.5 kb relative to the transcriptional start site of 17 089 human genes. ChIP-chip experiments were performed in duplicates. A variety of quality control metrics ensured high reproducibility and quality of the ChIP-chip data (see Materials and methods, Text S1, and Fig. S3). Regions bound by PARP3 were identified by applying criteria previously described [22]. In this way, we determined that PARP3 is bound to a set of 2205 regions (false discovery rate = 0.005) surrounding 1815 unique protein coding genes and 12 miRNAs by less than 10 kilobases. Given that the array is covering 17089 genes, PARP3 is associated with 11% of the genes present on the array. This is similar to the gene occupancy of other members of the PRC2 complex [22], [23].

**Figure 3:**
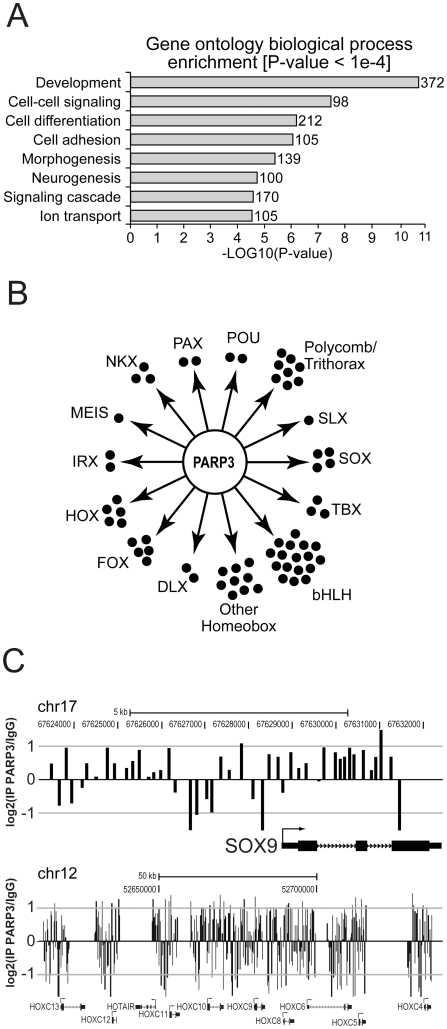
PARP3 targets developmental genes. A. Enrichment of PARP3 targets according to gene ontology annotations. B. Families of development-related transcription factors targeted by PARP3, as identified by ChIP-chip analysis. C. PARP3 ChIP-chip significant signals at SOX9 and HOXC loci. Rabbit IgG were used for the control ChIP. Transcription start sites are indicated by an arrow.

**Figure 4:**
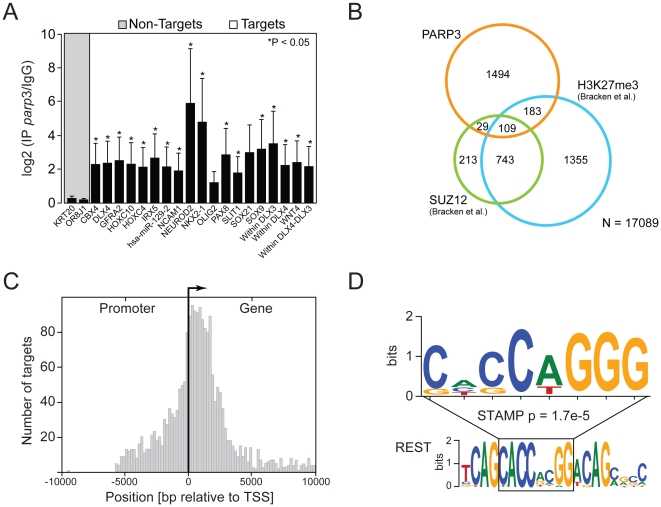
Analysis of PARP3-bound targets. A. Validation of PARP3 developmental targets by ChIP-qPCR. PARP3 ChIP were analyzed by standard qPCR using primers specific for regions targeted by PARP3 according to ChIP-chip results. Error bars represent the standard deviation from three independent experiments and asterisks indicate a significant enrichment relative to control (p<0.05). Two control regions (KRT20 and OR8J1), which are not bound by PARP3 (Non-targets), were used to determine basal signal. Several probes were used for DLX genes. The “DLX4” probe targets the promoter region, “within DLX” probes target sequences within the DLX genes and “within DLX3-DLX4” probe targets the DLX3-DLX4 intergenic region. B. Overlaps between PARP3 and SUZ12 targets or sequences enriched in H3K27me3. SUZ12 and H3K27me3 targets were those determined in human embryonic fibroblasts by [23]. C. Distribution of PARP3 target sequences relative to transcriptional start sites (TSS). D. PARP3 target sequences are enriched for the sequence CACCAGGG (upper sequence). This sequence matches part of the RE1-silencing transcription factor (REST) binding sequence (lower sequence).

Genes in the vicinity of PARP3-bound sequences were classified according to their biological processes using Gene Ontology (GO) annotations. We searched for enrichments in GO terms among PARP3-bound genes using the DAVID analysis tools [24]. This analysis revealed a remarkable enrichment of PARP3 around developmental genes (Fig. 3A). Many of these encode homeobox transcription factors regulating early specification events, including genes of the HOXC cluster, several members of the SOX, FOX, DLX, IRX families, as well as numerous genes encoding basic helix-loop-helix (bHLH) transcription factors (Fig. 3B; Fig. S4). Examples of probe signal distribution in genomic regions enriched for PARP3 is shown in Fig. 3C and Fig. S3. Collectively, these genes regulate axial and tissue patterning, cell fate specification, craniofacial development, and neurogenesis. Particularly, there is a striking enrichment for genes whose products are critical for neurogenesis, neural patterning, axon guidance, and synaptic communication such as the transcription factors SOX8/9/10/21, IRX5/6, MEIS3, NKX2-1/6-2, homeodomain-containing and several of the bHLH transcription factors, the axon guidance molecules SLIT1, SLIT3, KIRREL2, FEZ-1 and KAL1, synapse traffic and maturation related NPTX1 and NPTXR and the neural cell adhesion molecule NCAM1 among others (Fig. S4; Table S1). We also find that a number of genes encoding PcG and trxG proteins are PARP3 targets, including EZH1, a paralog of EZH2 [25], and MLL, a trxG important for the specification of neuronal lineage from neural progenitor cells [26]. Of note, PARP3 is also associated with HOTAIR, a long non-coding RNA transcribed from the HOXC cluster which has been proposed to recruit PcG at target genes [27], [28]. We also noticed that a number of PARP3 targets are part of the Wnt signaling pathway (WNT4, WNT7B, AXIN2, PYGO2, ASCL2, SFRP2, and BCL9) and of the FGF signaling pathway (FGF3 and FGFR3) (Fig. 4A; Table S1), two key signaling pathways during embryogenesis. Collectively, the analysis of PARP3 genomic occupancy supports the idea that PARP3 regulates transcription during development.

### Confirmation of PARP3 binding sites by quantitative PCR

Validation of PARP3-bound genes identified by ChIP-chip were conducted by ChIP followed by quantitative PCR (ChIP-qPCR) (Fig. 4A). We focused on PARP3-bound regions surrounding developmental genes given the impaired development of zebrafish embryos caused by reduced PARP3 expression. The ChIP-qPCR analyses (conducted independently from ChIP-chip experiments) revealed a significant and important enrichment of PARP3 on the majority of the selected genes relative to their enrichment after control ChIP with rabbit IgG. Maximal enrichment was observed around genes encoding the neurogenic differentiation 2 (*NEUROD2*), NK2 homeobox 1 (*NKX2.1*), in the coding region of the distal-less homeobox 3 (*DLX3*) gene and in proximity of sex determining region-Y box 9 gene (*SOX9*) (Fig. 4A). This set of data confirms that PARP3 does specifically bind developmental genes and in particular some crucial for neuronal specification.

### PARP3 targets are overlapping with SUZ12 and H3K27me3 targets

Given that some PARP3 associates with PRC2, we sought to compare genes bound by PARP3 with those associated with SUZ12 or enriched for H3K27me3. A significant overlap is found between PARP3-bound genes and PRC2 components (292 and 138 genes overlapping with H3K27me3 and SUZ12 respectively P<0.05) (Fig. 4B). This significant overlap is remarkable, considering SUZ12 and H3K27me3 bound genes were established in different cell lines, namely human embryonic fibroblasts [23]. The list of PARP3-associated genes overlapping with those bound by SUZ12 and H3K27me3 is given in Table S1. These observations strongly support an association between PARP3 and PRC2, and suggest that they could transcriptionally co-regulate development genes.

### PARP3 localization around transcription start site

We analyzed the distribution of PARP3 bound-regions relative to the transcription start site (TSS) of associated genes (Fig. 4C). To do so, we centered and/or reversed the orientation of every gene such that all of them start at the same position and point towards the same direction. We find that the distribution of PARP3 is preferentially downstream of the TSS rather than in the promoter region or at the TSS. The steep decrease in density at around 2500–3000 bp coincides with the end of the coverage of the Agilent promoter array. We noticed that the distribution of PARP3 spreads along several kilobase pairs of several target genes rather than being restricted to a small region. This blanket type distribution has also been observed for the PcG protein SUZ12 [22], [23].

### PARP3-bound regions are enriched for the REST binding sequence

PARP3-bound sequences were further examined for a potential enrichment in specific DNA sequences. The software Weeder was used to search through the 2205 DNA sequences bound by PARP3. A motif for which the consensus sequence is CACCAGGG was identified in 1164 sequences out of 2205 (53%) PARP3-bound regions (Fig. 4D). We interrogated the TRANSFAC database using STAMP [29] to determine whether this consensus corresponds to known binding motifs. Interestingly, this search identified RE1-silencing transcription factor (REST) as the closest transcription factor (P-value = 1.7e-5) that could potentially bind the identified sequence (Fig. 4D) [30]. REST is implicated in the self-renewal and pluritopency of embryonic stem cells [31] and acts as a repressor of neuronal gene expression in non-neuronal tissues [32]. This finding thus identifies a possible regulatory sequence through which PARP3 might regulate transcription, possibly in association with REST.

### Parp3 is an important regulator of neural crest cell specification

The identification of several PARP3 targets involved in development led us to assess whether they would be misregulated in zebrafish *parp3* morphants. Among genes potentially regulated by PARP3 in SK-N-SH cells, SOX9, DLX3 and DLX4 are of particular interest. During development of vertebrate embryos, these transcription factors are crucial in inducing early differentiation programs of neural crest progenitor cells into sensory placodes, oligodendrocytes, sensory neurons of the peripheral nervous system and pigment cells. In zebrafish embryos, orthologs of these genes (*sox9a* and *sox9b*, *dlx3b* and *dlx4b*) encode transcription factors crucial for the specification of non-neural ectoderm and neural crest cells into otic and olfactory placodes, pigment cells and the median fin fold [33], [34], [35]. An altered expression of these genes could therefore result in defective neural crest cell specification and migration, which could in turn induce the developmental caveats observed in zebrafish *parp3* morphants described above.

**Figure 5:**
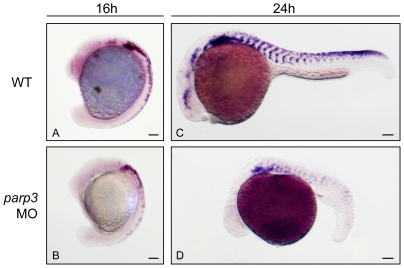
Expression of the neural crest cell marker *crestin* is impaired in *parp3* morphants. Zebrafish embryos were untreated (WT) or injected with 4 ng *parp3* MO1 and *crestin* expression was monitored by in situ hybridization. A. In 16 hpf WT embryos, *crestin* is expressed in premigratory neural crest cells and in neural crest cells migrating in the anterior trunk segments. B. In 16 hpf *parp3* morphants, *crestin* expression appears to be generally reduced. C. By 24 hpf, *crestin*-positive cells are distributed along the anterior-posterior axis, in neural crest migratory pathways of WT embryos. D. In 24 hpf *parp3* morphants, *crestin* expression is no longer detectable in the head and is markedly reduced in the trunk. Lateral views of embryos are shown with anterior to the left and dorsal to the top. Scale bars represent 10 µm.

By whole mount in situ hybridization (ISH), we first examined the formation and migration of neural crest cells in *parp3* morphants by monitoring the expression of the neural crest cell marker *crestin* [36] in zebrafish embryos with normal and reduced Parp3 expression. We find that the expression of *crestin* is indeed altered in *parp3* morphants (Fig. 5). At 16 hpf, *crestin* is normally expressed in premigratory neural crest cells and in migratory neural crest cells migrating from the most anterior trunk segments [36] (Fig. 5A). In *parp3* morphants however, the expression of crestin is generally reduced with most of the remaining expression limited to anterior trunk segments (Fig. 5B). *Crestin* expression is nearly undetectable in the hindbrain region. At 24 hpf, *crestin* expression could not be detected in the head and the tail regions, while the expression in the trunk of 24hpf-*parp3* morphants appears to be reduced (Fig. 5C, D). As crestin was shown to be expressed in all neural crest cells [36], our observations suggest a general perturbation in neural crest cell development in embryos with reduced Parp3 expression.

### Parp3 regulates sensory placode development in zebrafish embryos

**Figure 6:**
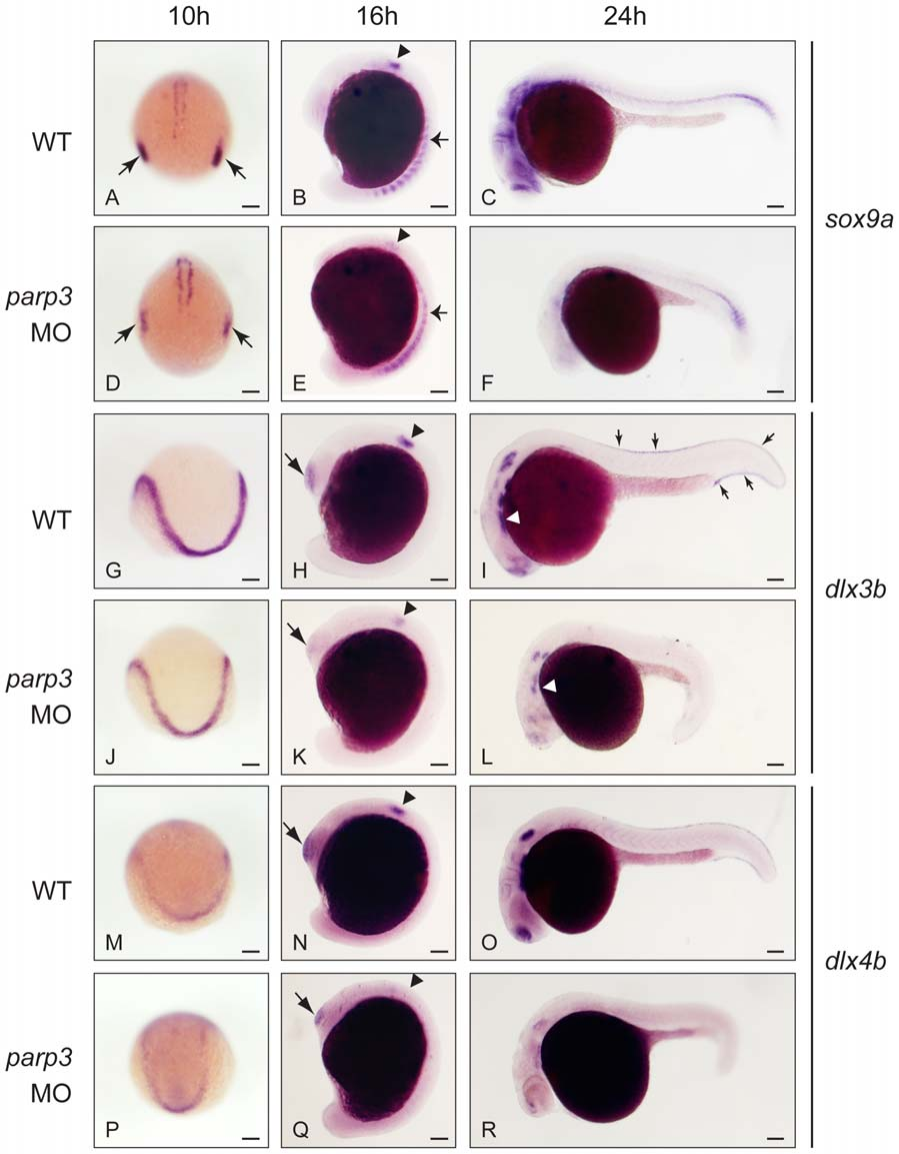
Impaired expression of *sox9a*, *dlx3b and dlx4b* in *parp3* morphants. Zebrafish embryos were untreated (WT) or were injected with 4 ng *parp3* MO1. Gene expression was detected by in situ hybridization. *A–F*. The expression of *sox9a* is drastically reduced in the otic placodes (small arrows) at 10 hpf and in the otic vesicles (arrowheads) at 16 hpf. Expression of *sox9a* in somite cells (small arrows in *B* and *E*) appears diffuse in *parp3* morphants. Expression of *sox9a* is almost completely abolished in the head region at 24 hpf (C, F). Expressions of *dlx3b* (*G–L*) and *dlx4b* (*M–R*) are minimally affected by *parp3* MO in ectodermal cells at 10 hpf (G, J, M, P) but are significantly reduced in the otic vesicles (arrowheads), olfactory placodes (large arrows) and branchial arches (white arrows) of *parp3* morphants at 16 hpf (H, K, N, Q) and 24 hpf (I, L, O, R). The expression of *dlx3b* and *dlx4b* is abolished in the median fin fold of 24 hpf *parp3* morphant embryos (small arrows in *I*). Dorsal views of embryos with anterior to the bottom in A, D, G, J, M, P and lateral views with anterior to the left, dorsal to the top, in B, C, E, F, H, I, K, L, N, O, Q and R. Scale bars represent 10 µm.

Having determined that *parp3* morphants have defective neural crest development, we next characterized the expression of *sox9a*, *dlx3b* and *dlx4b*. By ISH, we find that the expression of the neural crest specifier *sox9a* is indeed reduced in *parp3* morphants at 10, 16 and 24 hpf (Fig. 6A–F). The high level of expression of *sox9a* in the otic placode (Fig. 6A) and later in the otic vesicle (Fig. 6B) of wild type embryos is drastically reduced in *parp3* morphants (Fig. 6D, E). At 16 hpf, the expression of *sox9a* in the morphant paraxial cells is more diffuse suggesting that the somitogenesis is possibly disturbed (Fig. 6B, E). At 24 hpf, *sox9a* is normally expressed in the trunk and in three major areas of the head: the forebrain, the midbrain-hindbrain boundary and the pharyngeal arches (Fig. 6C). Expression in these three major sites is lost in the morphants and there is barely any expression in the trunk (Fig. 6F).

The *distal-less* related genes *dlx3b* and *dlx4b* encode homeobox transcription factors separated by a common short intergenic region that show largely overlapping expression patterns [33], [34], [37]. They are among the earliest transcription factors expressed in the otic and olfactory placodes, as well as in the median fin fold, a structure that is also affected in *parp3* morphants (Fig. 1C). Expression of *dlx3b* at the neural plate border is not severely affected in morphants at 10 hpf (Fig. 6G, J). However, *dlx3b* expression is almost completely lost in the otic vesicles of morphants at 16 hpf (Fig. 6H, K, arrowhead) and 24 hpf (Fig. 6I, L). At the same stage, there is also a decrease in *dlx3b* expression in the branchial arches (Fig. 6I, L, arrowhead). A reduced expression of *dlx3b* is also apparent in the olfactory placodes at 16 and 24 hpf (Fig. 6H, I, K, L). Finally, *dlx3b* expression in the median fin fold of 24h embryos (Fig. 6I, arrows) is nearly absent in the *parp3* morphants (Fig. 6L). Similar patterns of expression are observed with the *dlx4b* probe (Fig. 6M–R).

**Figure 7:**
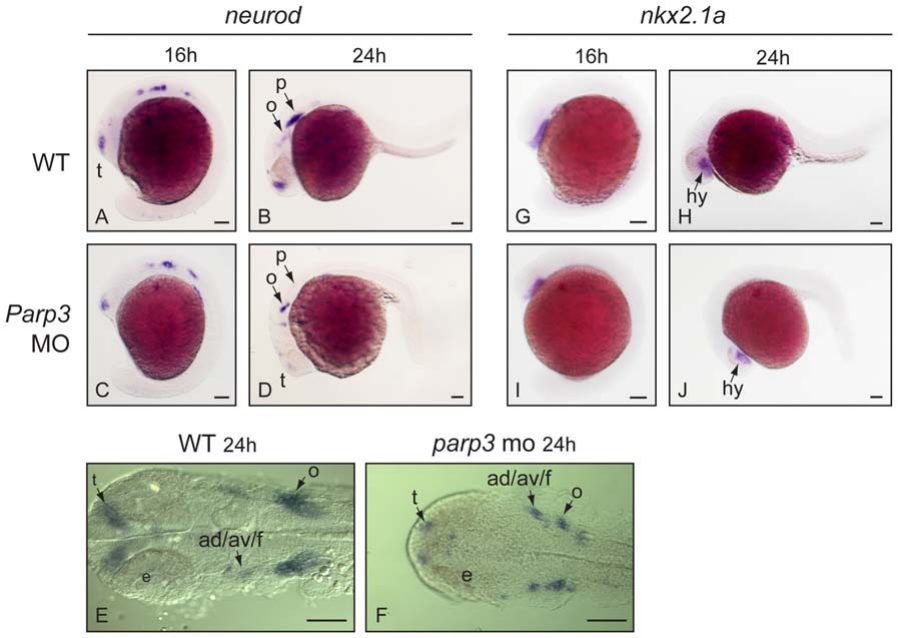
Expression of *neurod* and *nkx2.1* in *parp3* morphants. Expression of: A–F, *neurod* and G–J, *nkx2.1a* were determined in uninjected embryos or in embryos that received 4 ng of *parp3* MO1, by in situ hybridization. A–D and G–J are lateral views with anterior to the left, dorsal to the top. E–F are dorsal views of flat-mounted embryos with anterior to the left. ad/av/f: anterodorsal/anteroventral lateral line/facial placodes/ganglia; p: posterior lateral line placode; e: eye; o: octavel/statosacoustic ganglia precursors; t: telencephalon. Scale bars represent 10 µm.

The *neurod* and *nkx2.1a* genes are additional targets predicted from the genomic analysis of PARP3 distribution. The *neurod* gene encodes a bHLH transcription factor and is one of the earliest genes expressed in cranial placodes [38]. It plays a determinant role in the formation of the sensory placodes and the peripheral ganglia. The expression of *neurod* appears to be slightly reduced if at all in the trigeminal placode and in the anterior and posterior lateral line placode areas of 16 hpf *parp3* morphant embryos (Fig. 7A, B). However, by 24 hpf the overall expression of *neurod* is lower in the morphants than in wild type embryos. The most drastic reductions are seen in the telencephalon, in the octavel/statoacoustic placode, and in the posterior lateral line placode (Fig. 7B, D, E–F).

In zebrafish, expression of the NKX2.1 ortholog *nkx2.1a* is restricted to the ventral diencephalon (hypothalamus) of early embryos [39], while it is required for the proper development of the thyroid from the pharyngeal endoderm at later stages [40]. While the ChIP-chip analysis revealed that NKX2.1 is a target of PARP3, expression of *nkx2.1a* does not seem to be markedly affected during the first day of zebrafish development. The expression of *nkx2.1a*, which is mainly confined to the hypothalamus at 16–24hpf, is similar in wt and *parp3* morphant embryos (Fig. 7G–J).

Collectively, these observations clearly indicate that reduced Parp3 levels in zebrafish embryos impedes their development through an impaired expression of several transcriptional factors important for the specification of neural crest and sensory placode precursor cells.

## Discussion

The data presented here provide the first insights into the biological functions of the PARP family member PARP3. Using biochemical, genomic and *in vivo* approaches, we identify PARP3 as an important transcriptional regulator acting early in the development of sensory placodes and in the specification of neural crest cells of zebrafish embryos. Collectively, our findings suggest that PARP3 is an early key component in the regulation of the neural plate border formation in vertebrates.

The analysis of PARP3 genomic occupancy by ChIP-chip in the human SK-N-SH cells highlighted a predominant localization of chromatin-associated PARP3 around development genes, and in particular those involved in neurogenesis. Remarkably, an *in vivo* exploration, in the vertebrate animal model zebrafish, of PARP3 target genes identified in a human cell line revealed that the expression of several of these genes are indeed dependent on the expression of *parp3* during zebrafish embryonic development. The expression of *parp3* during zebrafish embryogenesis, which has been partially determined by ISH [41], is consistent with Parp3 exerting regulatory functions at these early stages. By the end of gastrulation (10 hpf), *parp3* is expressed at a basal level throughout the embryo with a stronger expression in the axis, while during segmentation (10h–16h), *parp3* expression is concentrated in the notochord. By 24 hpf, *parp3* expression is concentrated in the anterior/head region although not restricted to a specific structure. By knocking down the expression of Parp3 in zebrafish embryos, we readily observed a host of developmental defects within the first 48 hrs after fertilization consistent with impaired regulatory networks at the neural plate border, a region formed at the interface of the neuroectoderm and the non-neural ectoderm in the hindbrain of early embryos.

Under the influence of a specific set of transcription factors, multipotent precursor cells of the neural plate border give rise to the preplacodal ectoderm and to precursors of the neural crest cells. Subsequently, an important gene regulatory network orchestrates the formation, migration and differentiation of neural crest cells into cells of the parasympathetic nervous system, melanocytes, smooth muscle cells and craniofacial cartilage, among others [42], [43]. In parallel, the preplacodal ectoderm differentiates into sensory placodes from which are derived all of the cranial sensory ganglia. The SoxE family transcription factors SOX8, SOX9, SOX10 are critical neural crest “specifiers” and also direct proper differentiation of the preplacodal ectoderm together with DLX3 and DLX4 [35], [43]. The identification, in our ChIP-chip screen, of these genes as PARP3 targets therefore suggested that in zebrafish, Parp3 could participate in the specification of the neural plate border, neural crest formation and/or diversification by regulating the expression of these genes. Indeed, the significantly reduced expression of *crestin*, *sox9a*, *dlx3b*, *dlx4b* and *neurod*, the absence of inner ears (that take their origin in the preplacodal ectoderm), the delayed pigmentation (melanocytes are derived from neural crest cells) all support key transcriptional regulatory functions for Parp3 in the neural crest and neural plate border of early zebrafish embryos. Furthermore, because the expression of *sox9a* is already reduced by the end of gastrulation, our results suggest that parp3 is a critical determinant in the specification of the preplacodal ectoderm into otic placodes. Of note, a previous survey of PARP3 expression, in various tissues of adult monkeys, revealed a strong PARP3 expression in neurons of terminal ganglia, already suggesting that PARP3 may contribute to the functions of neurons of the peripheral nervous system [9]. As well, a recent study in a mouse model of multiple sclerosis showing a robust upregulation of PARP3 expression in the spinal cord of mice developing the symptoms of the disease also suggests a role for PARP3 in the central nervous system [44].

The reduced expression of *sox9a*, *dlx3b*/*dlx4b* and *neurod* in Parp3 zebrafish morphants indicates that PARP3 is required for maintaining normal expression levels of these key developmental genes. This observation was unexpected, in view of the association of PARP3 with PRC2 and the significant overlap between PARP3-bound and PcG-bound gene targets that rather suggested a repressor function for Parp3 (Fig. 4B). Furthermore, the regulation of *Sox9* by PRC2 in mouse ES cells has been recently demonstrated [15]. The relationship existing between PARP3 and PRC2 components may be context dependent, for example occurring in cells at a specific stage of lineage commitment, or in specific cell types. Nonetheless, given that only part of the nuclear PARP3 pool is associated with Polycomb bodies (Fig. 2B; [5]), our data could indicate that the transcriptional activation of *sox9a*, *dlx3b*, *dlx4b* and *neurod* by Parp3 is independent of its association with PRC2. Alternatively, it is conceivable that Parp3 could modulate the repression exerted by PRC2 at specific time points during development. In mouse ES cells, *Sox9* is in a bivalent domain, meaning that it is located in a region enriched for both the transcription repressive mark H3K27me3 and activating mark H3K4me3. Our limited understanding of bivalent genomic regions suggests that this epigenetic context allows maintaining the silencing of developmental regulators while at the same time keeping them ready for transcriptional activation at a later developmental stage [45], [46]. The regulated transition from fully silenced to fully expressed genes has been proposed to be mediated by proteins of the Trithorax complex and of PRC1 both in mouse ES cells and neural progenitor cells [26], [45], [46]. It is conceivable that PARP3 participates in the transition between the bivalent K4me3+K27me3 state to the activated K4me3 state. Our ChIP-chip data indicate that PARP3 could regulate the expression of the PRC2 components EZH1 and HOTAIR, as well as the trxG component MLL1. EZH1 is a paralog of EZH2 found in PRC2 complexes, HOTAIR is believed to regulate the binding of PRC2 to target sequences while MLL1 has been recently proposed to resolve bivalent chromatin marks, in particular at the Dlx2 gene during the specification of the neuronal lineage [25], [26], [28]. In addition, poly(ADP-ribosyl)ation of histones contributes significantly to the decondensation of chromatin [47]. Although mostly characterized in the context of PARP1-dependent poly(ADP-ribosyl)ation, histone H1 modification *in vitro* by PARP3 has been reported recently [6], [48] supporting the notion that PARP3 could also participate in chromatin remodelling at specific loci.

The identification of a consensus sequence in over half of PARP3-bound sequences that matches part of the REST binding site suggests that PARP3 could interact with many of its target sequences through another transcriptional regulatory complex comprising REST. REST binds a well defined consensus sequence together with several co-regulatory proteins including LSD1, CoREST, Sin3 and HDAC1/2. Previous studies have shown that REST represses the expression of neuronal specification genes in mouse ES cells, while differentiation of ES cells into neurons results in proteasomal degradation of REST and subsequent transcriptional activation of several target genes [49]. Furthermore, a possible co-regulation of transcription by REST and EZH2 has been put forward by a recent study in mouse ES cells in which a subset of bivalent chromatin domains occupied by EZH2 were found enriched for the REST consensus binding sequence [50]. Given that PARP3 interacts with HDAC1/2 and EZH2, it is possible that a subset of PARP3 could co-occupy and co-regulate genomic regions with REST during neuronal differentiation.

Among the PARP family, PARP3 is mostly related to PARP1 and PARP2, which form the type member subgroup of PARPs [18]. A number of studies have demonstrated that PARP1, PARP2, and poly(ADP-ribosyl)ation are important determinants for development [7], [51], [52]. Drosophila cannot develop beyond the larval stage when their unique *Parp* or *Parg* gene is mutated [51], [52]. Previous studies have shown that *Parp1* ^−/−^ and *Parp2* ^−/−^ mice develop normally but display a hypersensitivity to DNA damaging agents [7], [53]. However, the simultaneous knock-out of both genes in mice results in early embryonic lethality, revealing a functional redundancy between these two PARPs during DNA damage repair and during mouse development [7]. The lethality observed in *Parp1* ^−/−^/*Parp2* ^−/−^ mice further indicated that PARP3 cannot compensate for the absence of PARP1 and PARP2 during mouse development. Our study now reveals that Parp3 is essential for zebrafish development, implying that Parp1 and Parp2 cannot compensate for the biological functions of Parp3 during development and supporting the notion that Parp3 functions are distinct from those of Parp1 and Parp2 during vertebrate development.

Collectively, our work identifies PARP3 as an essential regulator of neurogenesis in vertebrates. Our data indicate that its functions are mediated through the positive regulation of several transcription factors key to the early specification of neural crest cells and sensory placodes. The developmental functions of PARP3 are distinct from those of PARP1 and PARP2 and may be linked to the epigenetic control exerted by Polycomb group proteins.

## Materials and Methods

### Cell culture

The human neuroblastoma cell line SK-N-SH (ATCC: HTB-11) was grown at 37°C in a 5% CO_2_ environment in DMEM (Invitrogen Corp.) supplemented with 1% Glutamax (Invitrogen), 10% foetal bovine serum (Wisent), 100 IU/ml penicillin and 100 µg/ml streptomycin (Invitrogen).

### Immunofluorescence, small-scale cellular fractionation, immunoprecipitation and immunoblotting

For immunofluorescence staining, SK-N-SH cells grown on coverslips were fixed in 4% formaldehyde diluted in PBS for 15 min, permeabilized for 5 min. in 0.5% Triton X-100 diluted in PBS and washed in PBS. Cells were incubated with rabbit polyclonal anti-PARP3 antibodies (1∶300; [5]) and mouse monoclonal anti-trimethylated K27 histone H3 (Abcam ab6002; 1∶300) for 90 min. at room temperature. Cells washed in PBS were then incubated with appropriate FITC- or Texas Red-conjugated secondary antibodies (Jackson ImmunoResearch) (1∶1000). Antibodies were diluted in PBS containing 10% FBS. Cells were photographed with a Zeiss AxioplanII motorized microscope equipped with a CoolSnapHQ cooled CCD camera.

Small-scale cellular fractionation was carried out as described [54]. SK-N-SH cells (2×10^7^) were harvested in PBS with a cell scrapper and washed twice in PBS. The cell pellet was resuspended in 500 µl buffer A (10 mM HEPES, pH 7.9; 10 mM KCl; 1.5 mM MgCl_2_; 0.34 M sucrose; 10% glycerol; 1 mM DTT; protease inhibitor cocktail (Roche)). Triton X-100 was added to the cells to a 0.1% final concentration and cells were incubated on ice for 8 min. Nuclei (fraction P1) were collected by a 5 min centrifugation at 1 300×*g*, 4°C. The supernatant S1 was spun 5 min at 20 000×*g*, 4°C to recover the cytoplasmic (S2) fraction. The nuclear P1 fraction was washed once in buffer A and lysed for 30 min on ice in 250 µl buffer B (3 mM EGTA; 0.2 mM EDTA; 1 mM DTT; protease inhibitor cocktail). Insoluble chromatin (P3) and soluble nuclear (S3) fractions were recovered by a 5 min centrifuation at 1 700×*g*, 4°C. P3 was washed once in buffer B, resuspended in Laemmli sample buffer and heated for 5 min. at 95°C. Samples were separated by SDS-PAGE, using a volume of each fraction corresponding to an equal cell number. They were transferred to PVDF membranes and probed for protein detection as described [5]. The following antibodies were used for protein detection: anti-EZH2 (1∶2500, clone 11, BD Pharmingen), anti-PARP1 (1∶5000; clone C_II_-10), anti-p38 (1∶2500, Cell Signaling), anti-PARP3 (1∶5000; rabbit polyclonal produced in-house [5]). Detection of Parp3 in zebrafish samples (Fig. 1A) was with a commercial rabbit polyclonal antibody (Enzo Life Sciences ALX-210-541; 1∶5000).

Immunoprecipitations of PARP3 from the nuclear (P3) fraction of SK-N-SH cells were carried out as described [5] using the commercial anti-PARP3 antibody (1 µl antibody/10^6^ cells). Rabbit IgG were used in control immunoprecipitations. Detection of immunoprecipitated proteins by immunoblotting was as described above.

### ChIP-chip analysis and data processing

ChIP were conducted as described [55]. PARP3 was immunoprecipitated from 3×10^7^ SK-N-SH cells with the commercial anti-PARP3 described above. In control ChIP, PARP3 antibodies were replaced by rabbit IgG. Two independent PARP3 and control ChIP-chip experiments were achieved. The DNA was amplified using LM-PCR and labelled with Cy5 (PARP3) and Cy3 (IgG) before hybridization on Agilent Human promoter arrays (two 244k chip per ChIP for a total of 4 chips). Detailed protocols can be found at http://www.ircm.qc.ca/microsites/francoisrobert/en. Regions significantly enriched for PARP3 relative to IgG were identified as described [22]. Briefly, the genome was scanned three probes at a time. When three probes were in a window of 1000 bp or less, they were further refered to as “triplets”. Triplets were considered significant when the PARP3 signal in 2 out of 3 probes had a p-value lower than 0.05 or when the center probe signal had a p-value lower than 0.01 and the first and last probes had a p-value lower than 0.1. Details of the bioinformatics analysis are given in Text S1. The false discovery rate is estimated at 0.005%. The assignment of triplets to the closest transcription start sites within a range of 10 000 bp was achieved using the UCSC genes of human build hg18. The ChIP-chip data shown within genomic context were extracted from our custom tracks at the UCSC Genome Browser (http://genome.ucsc.edu/). Raw and processed ChIP-chip data have been deposited in the Gene Expression Omnibus database (accession number GSE23709).

Gene set enrichment analyses were performed using the Database for Annotation, Visualization, and Integrated Discovery (DAVID) [24] using Biological Processes defined in Gene Ontology [56]. Gene set enrichments were considered significant when the p-value was lower than 1e-4.

### ChIP-qPCR

Primers for ChIP-chip confirmations were designed using the NCBI Primer-BLAST tool (http://www.ncbi.nlm.nih.gov/tools/primer-blast/). Sequences are given in Table S2. We designed pairs of primers within a selected subset of regions targeted and non-targeted by PARP3. ChIP independent from those for ChIP-chip were realized for the confirmatory quantitative PCR (qPCR). qPCR was performed in triplicate using SYBR green as described previously [55].

### Zebrafish analysis

Zebrafish embryos were obtained from in-house breeding of adults obtained at a local pet store and maintained using published methods [57]. All experiments were performed according to the guidelines of the Canadian Council on Animal Care and were approved by the University of Ottawa animal care committee (permit number BL-249). Detailed methods are described in Text S1. Morpholino oligonucleotides (MO) complementary to the translational start site of the zebrafish Parp3 (MO1) and to the sequence immediately upstream of the translational start site of Parp3 (MO2) had the following sequences: MO1: [5′ATGCTGCCCTTCTCTTGGGTGCCAT], MO2: [5′CTTTGTCCTCTGATACTGGCGGT-AC] (Fig. S1B). A non-targeting MO and a p53 MO used as controls had the following sequences: non-targeting MO: [5′ CCTCTTACCTCAGTTACAATTTATA 3′], p53 MO: [5′ GCGCCATTGCTTTGCAAGAAATTG]. All MO were obtained from Gene Tools Inc. One-cell wild-type embryos were microinjected with 1 nL of *parp3* MO (0.5 mM) using an IM 300 microinjector (Narishige). For the dose-response experiments (Fig. 1D), 1 nL of *parp3* MO were at the following concentrations: 0.25 mM (2 ng), 0.5 mM (4 ng), and 1.0 mM (8 ng). The p53 MO (0.5 mM) was microinjected alone or co-microinjected with *parp3* MO1 (0.5 mM) in a volume of 1 nL.

Whole mount in situ hybridization (ISH) was performed on 10, 16 and 24 hpf wt and *parp3* morphant embryos as described [58]. Fixed embryos were dehydrated in methanol and stored at −20°C until ISH. Anti-sense RNA probes corresponded to *dlx3b* (NM131322), *dlx4b* (NM131318), *sox9a* (NM161343), *crestin* (AF195881), *nkx2.1a* (NM131589), *neurod* (NM130978). Probes were synthesized from linearized cloned cDNAs using T7 or T3 RNA polymerase (Roche) and were conjugated with digoxigenin-UTP with the DIG RNA labeling mix (Roche) as described [58]. Embryos were progressively transferred in 100% glycerol prior to photography.

Embryos from the two transgenic fish lines fli:GFP and shh:GFP ABC#15 were kindly given by Dr M.-A. Akimenko (U. of Ottawa). They were injected at the 1-cell stage with parp3 MO1 and morphogenesis defects were examined under UV light.

## Supporting Information

Text S1 Supplemental materials and methods.
(DOC)

Table S1 PARP3 gene targets overlapping with Suz12 and H3K27me3 targets.
(DOC)

Table S2 Primers used for qPCR confirmation of PARP3 gene targets and non-targets.
(DOC)

Figure S1 A. Comparison of the amino acid sequences of the human PARP3 (accession number NP_005476) and zebrafish Parp3 (accession number NP_956795). The sequence in the WGR domain (green box) and the catalytic domain (blue box) is well conserved, including the WGR triad (small dots) and the residues critical for the poly(ADP-ribosyl)ation reaction H-Y-E (asterisks). B. Nucleotide sequence of zebrafish *parp3* and position of regions targeted by the morpholino oligonucleotides (**MO1** and **MO2**) used to attenuate parp3 expression in zebrafish. Only the 5′untranslated region (lower case) and the first 50 nucleotides of the coding region (uppercase) of the zebrafish *parp3* gene are shown (accession number NM200501).
(TIF)

Figure S2 Analysis of developmental defects in *parp3* morphants. A. Vasculature development in *parp3* morphants. Tg(fli1:EGFP)^y1^ zebrafish embryos were injected with *parp3* MO1. GFP is expressed exclusively in the vasculature. The vasculature development in *parp3* morphants is similar to that in control embryos, shown here at 48 hpf. B. Neural floor plate development in *parp3* morphants. One-cell embryos from transgenic zebrafish expressing GFP under the control of the sonic hedgehog (*shh*) promoter were injected with *parp3* MO1. GFP is expressed specifically in the floor plate. Despite the highly curved trunk in *parp3* morphants, the neural floor plate pattern is similar to that of control embryos, shown at 48 hpf. C. Motoneuron development in *parp3* morphants. The distribution of synaptic vesicle 2 (sv2), a marker of motoneurons, was monitored to determine if ill-developed motoneurons could explain the impaired motility of morphants. Wild type zebrafish embryos injected or not with *parp3* MO1 were fixed at 24 hpf and immunostained with an anti-sv2 antibody. Lower images represent higher magnification views of the trunk region. Motoneurons appear to develop normally in *parp3* morphants. Embryos were visualized under a fluorescence microscope. Scale bars represent 10 µm.
(TIF)

Figure S3 ChIP-chip analysis and data processing. Probe signal intensity obtained in each replicate is given for genomic regions comprising the target genes DLX3/4, SOX9 and of the HOXC cluster and the non-target genes encoding olfactory receptors and keratins. There is a very good correlation between the replicates. Significant binding of PARP3 is detected for DLX3/4, SOX9 and HOXC loci but not for olfactory receptors and keratin loci shown.
(TIF)

Figure S4 Detailed representation of Figure 3B. Represented genes correspond to those identified as PARP3 target genes by ChIP-chip that encode transcription factors involved in the regulation of development.
(TIF)

Movie S1 Reduced motility of *parp3* morphants. A zebrafish embryo injected with 4 ng *parp3* MO1 at the one cell stage was filmed 48 hours post-fertilization.
(MPG)
