# Supplementary material for: A Key Role for Poly(ADP-Ribose) Polymerase 3 in Ectodermal Specification and Neural Crest Development
Source: PLoS One. 2011 Jan 17;6(1):e15834. doi: 10.1371/journal.pone.0015834 (PMC3022025; doi:10.1371/journal.pone.0015834)
Supplement: Text S1 — Supplemental materials and methods. (DOC) [file pone.0015834.s001.doc]

Supplemental Material

***Zebrafish.*** Embryos were grown in 1X embryo media as described [1]. Embryos and larvae were manually dechorionated and/or deyolked at specified times post-fertilization and processed for immunoblot analysis or fixed in 4 % paraformaldehyde (PFA)/phosphate buffered saline (PBS) at 4°C overnight for in situ hybridization (ISH) or immunostaining. For immunoblot analysis of zebrafish proteins, 25 embryos were solubilized in 50 μl Laemmli sample buffer containing 4M urea. They were lyzed by sonication (3 cycles of 15 seconds using the microtip of a Sonic dismembrator 550) and heated at 65°C for 15 min. Extracts were clarified by a 10 min. centrifugation at 16 000 *x g*. Immunoblots were as described in Materials and Methods.

Immunostaining was carried out on 24 hpf wt or *parp3* morphant embryos. Fixed embryos were washed 3 times in PBS containing 0.1% Tween-20 (PBS-T) and incubated for 2 h at 4 ºC in blocking solution (0.5 % Triton X-100, 4% calf serum and 1% DMSO in PBS). Embryos were washed in PBS-T prior to an overnight incubation at 4 °C with the SV2 antibody (1:200 in 1X PBS; Developmental Studies Hybridoma Bank) kindly given by Dr. M. Jonz (U. of Ottawa). Embryos washed in PBS-T were then incubated with a goat anti-mouse antibody conjugated to AlexaFluor 594 (1:100 in PBS). Visualization of stained embryos with a Nikon SMZ1500 microscope was under UV light.

***Bioinformatics and statistical analyses*.** The Agilent chips were scanned using an Axon 4000B scanner and processed with Genepix Pro 4.1. The data were analyzed in R (http://www.r-project.org/) using the package limma in BioConductor [2]. An excellent correlation is observed between probe signals at selected loci (Fig. S3). The background was subtracted from the raw signal and the logarithmic 2 is used for median normalization. In addition to the analysis of probe signals in triplets as described in Materials and Methods, another criteria was applied to each triplet to identify significant ones. A score was calculated on the basis of the mean value of the minus logarithm 10 of the 3 probe p-values. This score was then compared to scores obtained by all the possible valid triplets generated at random by permuting 20 times the p-values of all the probes on the array. Using this technique, it was possible to estimate the null distribution and set a cutoff such that only 0.005 % of the random triplets have a score higher than this cutoff, which yield an estimated FDR of 0.005. If the triplet score is higher than the cutoff, we considered this region to be bound by PARP3. We then linked contiguous regions of triplets to form a single region. The assignment of triplets to the closest transcription start sites within a range of 10000 bp was achieved using the UCSC genes of human build hg18.

Statistical analyses were performed using R (<http://www.r-project.org/>). The significant difference between IgG and PARP3 immunoprecipitations in ChIP-qPCR confirmation (Fig. 4A) was established from the raw CTs using a two-sided Student’s t-test with equal variance.

The Venn diagram displayed in Fig. 4B was generated using the online tool BioVenn [3]. The significance of the overlap between this study and the one of Bracken et al. displayed in Fig. 4B was assessed using a Fisher Exact test with a total number of 17089 genes which is the total number of unique official gene symbols overlapping between the Nimblegen and Agilent Promoter array platforms.

Genomic sequences used for the motif enrichment analysis were extracted from the hg18 human build using Galaxy [4]. The DNA motif enriched in regions bound by PARP3 was identified using Weeder [5] and the following command : “weederlauncher.out file.fasta HS large ST10”. The logo of the motif identified as enriched within the regions bound by PARP3 was drawn using WebLogo [6].

References

1. Nüsslein-Volhard C, Dahm R, editors (2002) Zebrafish: Oxford University Press. 328 p.

2. Smyth GK (2004) Linear models and empirical bayes methods for assessing differential expression in microarray experiments. Stat Appl Genet Mol Biol 3: Article3.

3. Hulsen T, de Vlieg J, Alkema W (2008) BioVenn - a web application for the comparison and visualization of biological lists using area-proportional Venn diagrams. BMC Genomics 9: 488.

4. Taylor J, Schenck I, Blankenberg D, Nekrutenko A (2007) Using galaxy to perform large-scale interactive data analyses. Curr Protoc Bioinformatics Chapter 10: Unit 10 15.

5. Pavesi G, Mereghetti P, Zambelli F, Stefani M, Mauri G, et al. (2006) MoD Tools: regulatory motif discovery in nucleotide sequences from co-regulated or homologous genes. Nucleic Acids Res 34: W566-570.

6. Crooks GE, Hon G, Chandonia JM, Brenner SE (2004) WebLogo: a sequence logo generator. Genome Res 14: 1188-1190.
