# Supplementary material for: A Key Role for Poly(ADP-Ribose) Polymerase 3 in Ectodermal Specification and Neural Crest Development
Source: PLoS One. 2011 Jan 17;6(1):e15834. doi: 10.1371/journal.pone.0015834 (PMC3022025; doi:10.1371/journal.pone.0015834)
Supplement: Table S1 — PARP3 gene targets overlapping with Suz12 and H3K27me3 targets. (DOC) [file pone.0015834.s002.doc]

Table S1: PARP3 gene targets overlapping with Suz12 and H3K27me3 targets*

| PARP3-SUZ12-H3K27me3 | PARP3-SUZ12 | PARP3-H3K27me3 |
| --- | --- | --- |
| ACCN1  AQP5  B4GALNT2  C13orf18  C1QL4  C20orf114  C21orf29  C22orf33  C4orf31  CABP7  CACNA1A  CAMKV  CASKIN1  CCND2  CHN2  CHRDL1  CKM  CLDN9  CNFN  COCH  DIO3OS  DLK1  DLX3  DLX4  DSCAML1  DUSP9  FAM57B  FAM70A  FAM83F  FBXL16  FGF3  GALNTL1  GFRA1  GFRA2  GJB4  GNA14  GPR97  GRIN2C  GRIN2D  HOXC10  HOXC4  HOXC5  IGF2  IRF5  IRF8  JPH3  JPH4  KAL1  KCNK10  KIAA0574  KIRREL2  KL  KRT4  LBX1  LINGO1  LMX1B  LOR  MEGF11  MLN  MSI1  MT1A  NDRG4  NEFH  NEURL  NKX2-1  NKX2-5  NPAS4  NTRK3  OLIG2  OTOP2  PCK1  PHOX2A  PKD2L1  PLEKHA7  PNPLA5  PPP1R1A  PPP1R1B  PRSS22  PTCHD1  PTPN3  PYY  RASGRP2  RDH16  RET  RHBDL3  RTN4RL2  SAG  SFRP2  SHF  SLC17A7  SLC26A10  SLC6A2  SLC7A10  SLIT1  SMTNL2  SOX21  SSTR2  SULF2  SYT3  TCF15  TERT  TFAP2C  TMC6  TMEM132E  TNFAIP2  TNK1  VAX2  VIPR1  VMO1 | AANAT  ALDH3A1  ALPI  APLP1  APOBEC3A  AQP2  ARHGDIG  ARHGEF15  ASCL2  ATP12A  ATP1A3  ATP2B3  ATP4A  AXIN2  BEGAIN  C14orf166B  C16orf67  C1orf65  C20orf112  C20orf151  C20orf185  CABP2  CACNG1  CALCB  CCDC48  CCL17  CD8B  CD93  CDH23  CEACAM7  CERCAM  CHP2  CHRM1  CHRNA4  CHRND  CHST1  CLDN5  CLEC10A  CNGB1  CPNE6  CREB3L3  CSF3R  CSTL1  CXCL16  CYP2F1  DAGLA  DEFB124  DHRS2  DLGAP4  DPEP1  DRD2  DTX1  EDAR  EEF1A2  EGFL7  ENTPD3  ERAF  FBN3  FCER2  FCGBP  FRMPD2  FSCN2  FXYD7  GABBR2  GALR3  GCK  GDF10  GGT6  GPR35  GPR68  GPR81  GRIK5  GUCA2B  HBM  HCG9  HRC  HRH3  HSPA12B  IGFBP1  IL29  IRX5  ISLR  ITGB2  ITIH1  JAG2  KCNC3  KCNQ1  KCNS2  KIAA1984  KLK1  KLK15  KLK2  KNCN  KRT18  KRT35  KRT5  KRT75  KRT77  KRT82  L1CAM  LMAN1L  LSP1  LYPD2  MAST1  MIP  MOGAT2  MPO  MRGPRD  MSLN  MYH6  MYO15B  NAALADL1  NCAM1  NCF4  NCR2  NEUROD2  NFAM1  NGB  NIPA1  NMUR1  NPBWR2  NPTXR  OBSCN  PADI1  PADI2  PAPLN  PARVG  PAX8  PCDH1  PDE2A  PDE4C  PIPOX  PLA2G3  PLA2G4E  POU2F2  PPP1R16B  PRF1  PRKCG  PTPRN  PYY2  RAI2  RASGEF1C  RORC  SAMD11  SCRT2  SEPT3  SH2D4B  SIGLEC1  SIGLEC6  SIGLEC7  SLC12A5  SLC14A2  SLC22A8  SLC26A9  SLC29A2  SLC47A2  SLC8A2  SLCO4A1  SLIT3  SMPD3  SPDEF  SPTBN2  STAC2  STRA6  SYT8  TAC3  TBX4  TEX101  THBD  THEM5  TM4SF5  TMC8  TMEM174  TMPRSS5  TNFRSF14  TNNT3  TRIM29  TRPM2  TSPAN18  TUB  TULP1  UBQLN3  USH1C | C9orf127  CBX4  COASY  CPLX1  CST11  DCST2  GPR133  HK1  IZUMO1  LILRB5  MED13L  MN1  MPPED1  MTMR1  NKX6-2  NPTX1  NSBP1  NYX  OR10P1  PEMT  PLCB1  POLG  PSD4  SALL2  SLC38A5  SLC7A4  TAF15  TM9SF4  UNC45B |

*Genomic occupancy of SUZ12 and H3K27me3 was determined in the human embryonic diploid fibroblast cell line TIG3 by {Bracken, 2006 #16}.
