# Supplementary material for: A Key Role for Poly(ADP-Ribose) Polymerase 3 in Ectodermal Specification and Neural Crest Development
Source: PLoS One. 2011 Jan 17;6(1):e15834. doi: 10.1371/journal.pone.0015834 (PMC3022025; doi:10.1371/journal.pone.0015834)
Supplement: Table S2 — Primers used for qPCR confirmation of PARP3 gene targets and non-targets. (DOC) [file pone.0015834.s003.doc]

Table S2: Primers used for qPCR confirmation of PARP3 targets and non-targets
