# Supplementary figures and images for: A Key Role for Poly(ADP-Ribose) Polymerase 3 in Ectodermal Specification and Neural Crest Development
Source: PLoS One. 2011 Jan 17;6(1):e15834. doi: 10.1371/journal.pone.0015834 (PMC3022025; doi:10.1371/journal.pone.0015834)

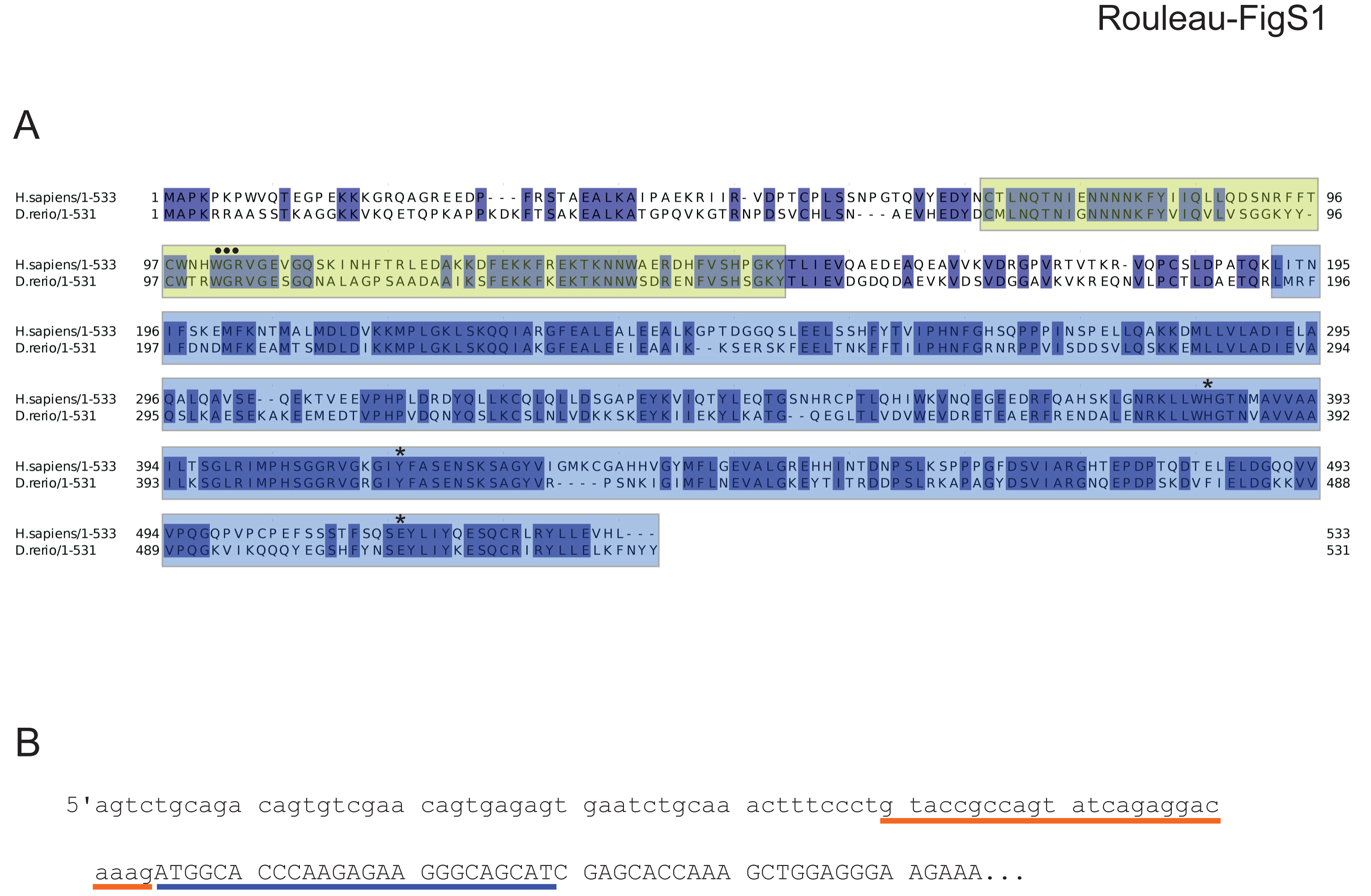

Supplement: Figure S1 — A. Comparison of the amino acid sequences of the human PARP3 (accession number NP_005476) and zebrafish Parp3 (accession number NP_956795). The sequence in the WGR domain (green box) and the catalytic domain (blue box) is well conserved, including the WGR triad (small dots) and the residues critical for the poly(ADP-ribosyl)ation reaction H-Y-E (asterisks). B. Nucleotide sequence of zebrafish parp3 and position of regions targeted by the morpholino oligonucleotides (MO1 and MO2) used to attenuate parp3 expression in zebrafish. Only the 5′untranslated region (lower case) and the first 50 nucleotides of the coding region (uppercase) of the zebrafish parp3 gene are shown (accession number NM200501). (TIF) [file pone.0015834.s004.tif]

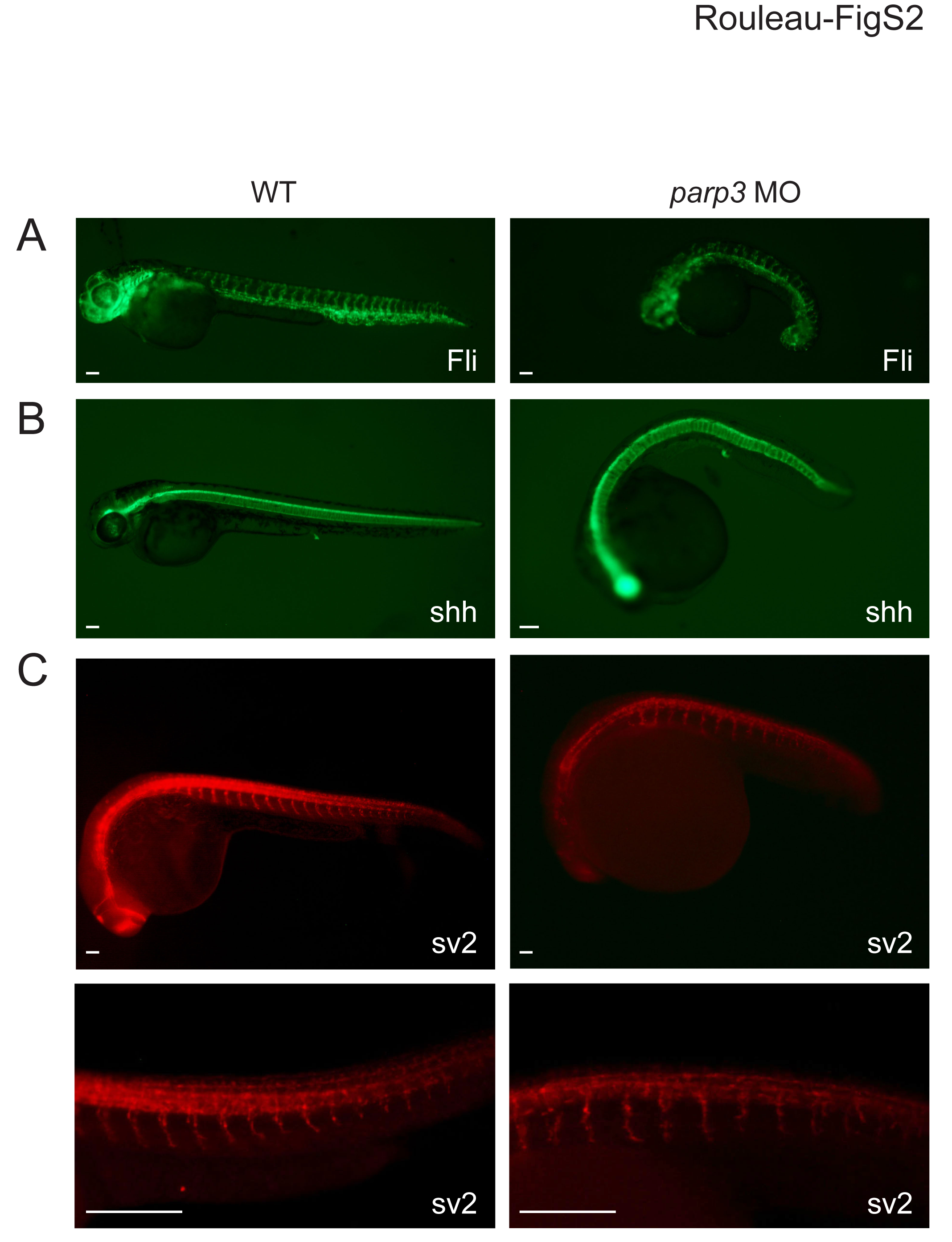

Supplement: Figure S2 — Analysis of developmental defects in parp3 morphants. A. Vasculature development in parp3 morphants. Tg(fli1:EGFP)y1 zebrafish embryos were injected with parp3 MO1. GFP is expressed exclusively in the vasculature. The vasculature development in parp3 morphants is similar to that in control embryos, shown here at 48 hpf. B. Neural floor plate development in parp3 morphants. One-cell embryos from transgenic zebrafish expressing GFP under the control of the sonic hedgehog (shh) promoter were injected with parp3 MO1. GFP is expressed specifically in the floor plate. Despite the highly curved trunk in parp3 morphants, the neural floor plate pattern is similar to that of control embryos, shown at 48 hpf. C. Motoneuron development in parp3 morphants. The distribution of synaptic vesicle 2 (sv2), a marker of motoneurons, was monitored to determine if ill-developed motoneurons could explain the impaired motility of morphants. Wild type zebrafish embryos injected or not with parp3 MO1 were fixed at 24 hpf and immunostained with an anti-sv2 antibody. Lower images represent higher magnification views of the trunk region. Motoneurons appear to develop normally in parp3 morphants. Embryos were visualized under a fluorescence microscope. Scale bars represent 10 µm. (TIF) [file pone.0015834.s005.tif]

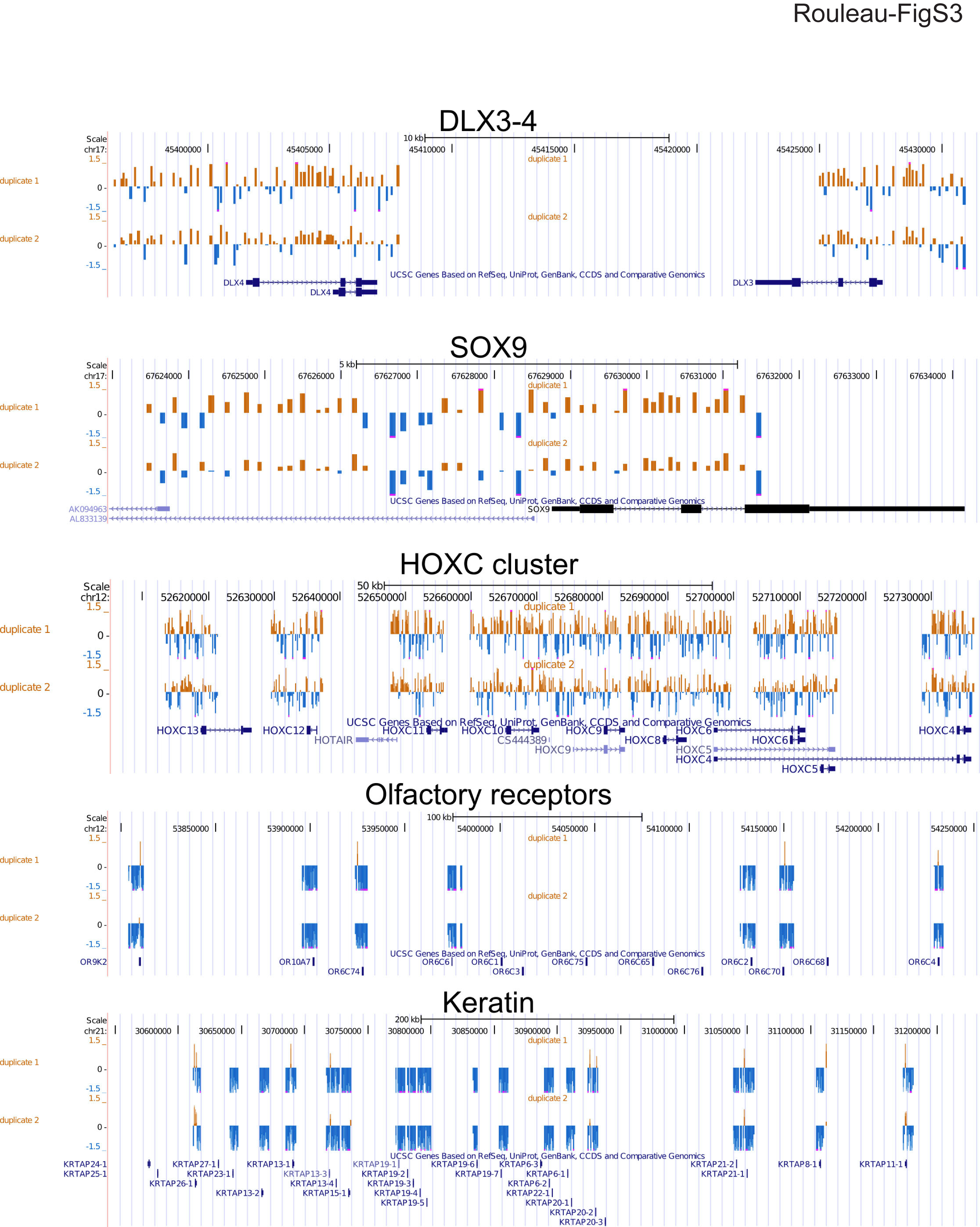

Supplement: Figure S3 — ChIP-chip analysis and data processing. Probe signal intensity obtained in each replicate is given for genomic regions comprising the target genes DLX3/4, SOX9 and of the HOXC cluster and the non-target genes encoding olfactory receptors and keratins. There is a very good correlation between the replicates. Significant binding of PARP3 is detected for DLX3/4, SOX9 and HOXC loci but not for olfactory receptors and keratin loci shown. (TIF) [file pone.0015834.s006.tif]

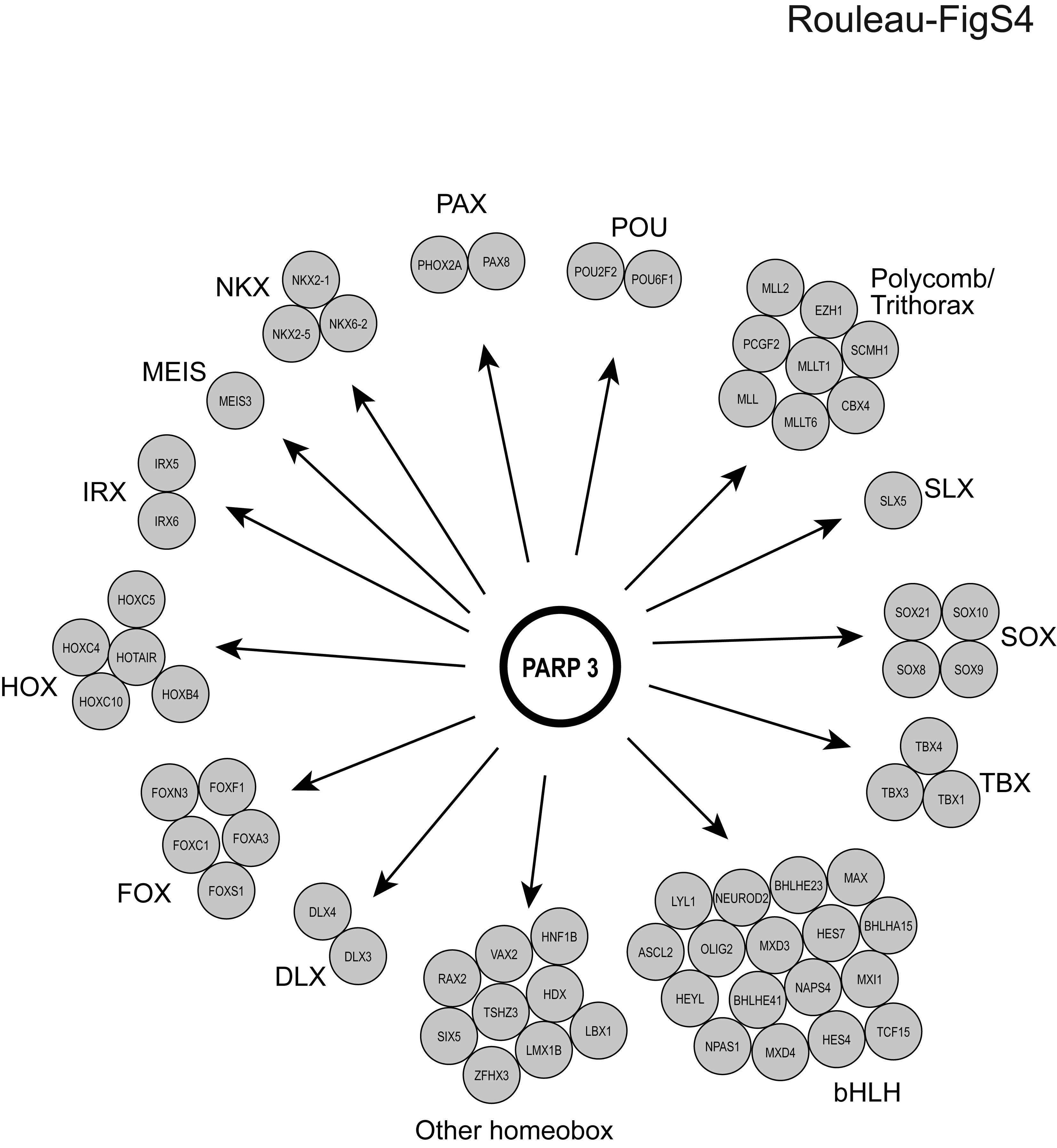

Supplement: Figure S4 — Detailed representation of Figure 3B. Represented genes correspond to those identified as PARP3 target genes by ChIP-chip that encode transcription factors involved in the regulation of development. (TIF) [file pone.0015834.s007.tif]
